# Immunoglobulins concentration in HIV-infected patients' with viral hepatitis, candidiasis and herpes simplex viral infection

**DOI:** 10.1186/1742-4690-9-S1-P148

**Published:** 2012-05-25

**Authors:** Narina Sargsyants, Tigran Davtyan

**Affiliations:** 1 Armenicum Clinical Center, Yerevan, Armenia

## Introduction

Coinfection with viral hepatitis (VH) and some opportunistic infections (OI) can decrease HIV-infected patients' survival. The purpose of this study was evaluation of humoral immunity factors in the commonest concomitant and OI.

## Materials and methods

110 HIV-infected patients were involved in the study. Among them 76% had candidiasis, 74% VH, 23% HSV-infection. Determination of IgG, IgM, and IgA was performed by immune turbidimetry method by using Human test kits, Germany. Statistic analyses were performed by parametric and non-parametric procedures for paired samples T-test.

## Results

**Figure 1:**
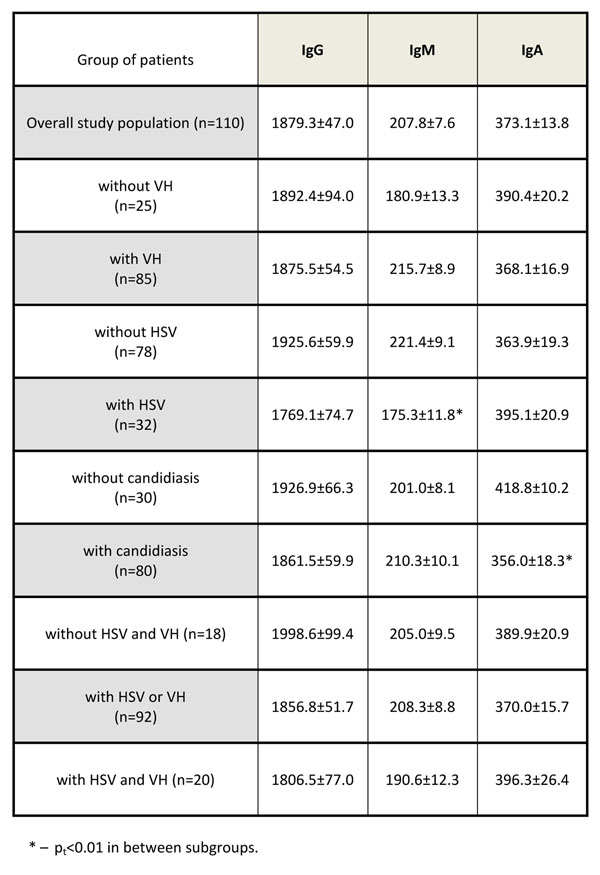


There was no significant difference in serum IgG levels between overall study population and individual patient groups. The most significant difference in IgM concentrations was found in the group with HSV. Thus, IgM level in patients with HSV was significantly lower in comparison with group without HSV (p=0.014) as well as with patients with both HSV and VH (p=0.029). IgA level was higher in patients without candidiasis (p=0.022) in comparison with general study population. With purpose to clarifying the degree of intra-group heterogeneity in Ig parameters patient were additionally divided into subgroups. It was revealed that IgG concentration in patients with oral candidiasis (1663±59 mg/dl, n=32) was different from that in the patients with not only oral candidiasis, but other localizations (1945±89 mg/dl, n=48, p=0.05). It is important to mention that higher IgM и IgG were enrolled in VH with moderate and high activity of ALT and AST. Figure 1

## Conclusions

Difference in humoral immunity disorders in HIV-infection depend on certain associated infections as well as on their severity.

